# Tumor necrosis factor inhibitors are associated with a decreased risk of COVID‐19‐associated hospitalization in patients with psoriasis—A population‐based cohort study

**DOI:** 10.1111/dth.15003

**Published:** 2021-06-05

**Authors:** Khalaf Kridin, Yochai Schonmann, Giovanni Damiani, Avi Peretz, Erez Onn, Dana Tzur Bitan, Arnon D. Cohen

**Affiliations:** ^1^ Lübeck Institute of Experimental Dermatology University of Lübeck Lübeck Germany; ^2^ Azrieli Faculty of Medicine Bar‐Ilan University Safed Israel; ^3^ Clalit Health Services Tel‐Aviv Israel; ^4^ Clinical Dermatology IRCCS Istituto Ortopedico Galeazzi Milan Italy; ^5^ Department of Biomedical, Surgical and Dental Sciences University of Milan Milan Italy; ^6^ Department of Pharmaceutical and Pharmacological Sciences University of Padua Padua Italy; ^7^ Baruch Padeh Medical Center Tiberias Israel; ^8^ Department of Behavioral Sciences Ariel University Ariel Israel; ^9^ Sial Research Center, Division of Community Health Ben Gurion University of the Negev Beer Sheva Israel

**Keywords:** infection‐ bacterial/fungal/viral, psoriasis

## Abstract

The risk of coronavirus disease 2019 (COVID‐19) and its complications among patients with psoriasis treated by tumor necrosis factor inhibitors (TNFis) remains to be decisively delineated. We aimed to assess the risk of COVID‐19 infection, COVID‐19‐associated hospitalization, and mortality among Israeli patients with psoriasis treated by TNFi relative to other systemic agents. A population‐based cohort study was conducted to compare psoriasis patients treated by TNFi (*n* = 1943), with those treated by methotrexate (*n* = 1929), ustekinumab (*n* = 348), and acitretin (*n* = 1892) regarding COVID‐19 outcomes. Risk of investigated outcomes was assessed using uni‐ and multi‐variate Cox regression analyses. The incidence rate of COVID‐19, COVID‐19‐associated hospitalization, and mortality in the TNFi group was 35.8 (95% CI, 26.1‐47.9), 0.8 (95% CI, 0.0‐4.2), and 0.0 per 1000 person‐years, respectively. Exposure to TNFi was associated with a comparable risk of COVID‐19 infection [adjusted hazard ration (HR) for TNFi vs methotrexate: 1.07 (95% CI, 0.67‐1.71); TNFi vs ustekinumab: 1.07 (95% CI, 0.48‐2.40); TNFi vs acitretin: 0.98 (95% CI, 0.61‐1.57)]. TNFi was associated with a decreased risk of COVID‐19‐associated hospitalization relative to methotrexate (adjusted HR, 0.10; 95% CI, 0.01‐0.82) and ustekinumab (adjusted HR, 0.04; 95% CI, 0.00‐0.64), but not to acitretin (adjusted HR, 1.00; 95% CI, 0.16‐6.16). No significant difference in COVID‐19‐associated mortality was found between the four different groups. TNFi was associated with a decreased risk of admissions due to COVID‐19. Our findings substantiate the continuation of TNFi treatment during the pandemic. TNFi may be positively considered in patients with moderate‐to‐severe psoriasis warranting systemic treatment during the pandemic.

## 1 INTRODUCTION

Given that severe coronavirus disease 2019 (COVID‐19) is associated with a hyperinflammatory state,^1^, ^2^ it is highly pertinent to investigate whether the presence of preexisting immune‐mediated diseases or the previous exposure to immunomodulatory drugs affect the manifestations of COVID‐19. While several studies displayed an increased risk and more aggressive course of SARS‐CoV‐2 infection in patients with certain immune‐mediated and inflammatory diseases,^3^, ^4^, ^5^ others disproved these observations.^6^, ^7^, ^8^, ^9^, ^10^

Tumor necrosis factor inhibitor (TNFi), the largest drug class worldwide,^11^ was proved efficacious in a wide array of autoimmune and inflammatory diseases.^12^ While TNFi has dramatically improved outcomes of patients with moderate‐to‐severe plaque psoriasis, it is implicated with different infections.^13^ While the use of this immunomodulatory drug during the current pandemic raised many concerns, recent observational studies ascribed a favorable protective role for TNFi among patients with inflammatory bowel disease (IBD) and rheumatic diseases who developed COVID‐19.^4^, ^14^ In psoriasis, however, the risk of COVID and its complications among patients undergoing TNFi treatment remains to be delineated. The current knowledge about the safety of immunomodulatory drugs in psoriasis stems from observational studies of short follow‐up durations and lacks head‐to‐head comparison between different agents, as most former studies pooled patients under ''biologics'' or ''immunomodulatory drugs'' together.^15^, ^16^, ^17^, ^18^, ^19^

In the current study, we sought to investigate the risk of COVID‐19 infection, COVID‐19‐associated hospitalization, and mortality among patients with psoriasis treated by TNFi (adalimumab, etanercept, and infliximab). To precisely assess the safety of this treatment during the pandemic, patients on TNFi were compared with three reference groups: (i) psoriasis patients treated by methotrexate, (ii) ustekinumab, and (iii) acitretin. A sensitivity analysis was held to dissect the outcomes under each one of the three investigated TNF agents.

## 2 METHODS

### 2.1 Study design and dataset

We performed a historical retrospective cohort study that followed patients with psoriasis to assess the incidence of COVID‐19, COVID‐associated hospitalization, and mortality. The study was approved by the institutional review board (IRB) in accordance with the declaration of Helsinki (approval code: 0212‐17‐COM).

The current study was based on the computerized database of Clalit Health Services (CHS). CHS is the largest healthcare maintenance organization in Israel, covering a wide variety of private and public healthcare services for 4 540 768 enrollees as of October 2018. CHS database is typified by an inclusive nature owing to its ability to retrieve data from numerous sources originating both from ambulatory and hospitalized care settings. CHS is additionally eminent in negligible loss to follow‐up and free access to healthcare services. All these features render the CHS database highly compatible with the conduction of reliable and robust epidemiological analyses.^20^

### 2.2 Study population

The computerized database of CHS was systematically screened for all prevalent cases with a diagnostic code of psoriasis as registered by board‐certified dermatologists. Eligible patients had to be alive and active members of CHS at the onset of the pandemic, defined as the date of the first confirmed case of COVID‐19 in Israel (February 27th, 2020).

### 2.3 Definition of exposure and different analyses

Exposure to all drugs was defined in case the drug was prescribed for at least 1 month during the pandemic. For the main analysis, patients with psoriasis receiving TNFi were compared to those receiving methotrexate as a reference group. Methotrexate was selected as a referent drug given that it is the most commonly prescribed nonbiologic systemic therapy for psoriasis,^21^ and in accordance with other international registry‐based studies.^22^ Patients managed by TNFi or methotrexate in conjunction with other concomitant systemic immunomodulatory/immunosuppressive drugs were excluded from the analysis enabling to evaluate the independent influence of the drug of interest. Concomitant drugs warranting exclusion were: cyclosporine, interleukin (IL)‐17 inhibitors, ustekinumab, and apremilast. Concomitant administration of retinoids did not represent an exclusion criterion.

A sensitivity analysis was performed to assess the risk of COVID‐19 outcomes in patients treated by the three widely utilized TNFi agents in Israel; adalimumab, etanercept, and infliximab. Each of these drugs was separately compared to methotrexate. Numerous patients were exposed to more than a single TNFi agent during the pandemic. The time under each of the drugs was calculated separately in the respective sensitivity analysis, whilst the cumulative time under the various drugs was considered in the main analysis.

The secondary analysis evaluated the risk of COVID‐19 and its complication in patients managed by TNFi relative to those receiving (i) ustekinumab and (ii) acitretin. Since concomitant exposure to acitretin did not merit exclusion from the primary analysis, all patients treated concomitantly by TNFi and acitretin were excluded only from the secondary analysis comparing TNFi vs acitretin.

### 2.4 Definition of COVID‐19‐related outcomes

The medical records of eligible patients were checked for a diagnosis of COVID‐19. The diagnosis of COVID‐19 was based on confirmation of cases by US FDA‐approved molecular tests. COVID‐19‐associated hospitalization was defined in COVID‐19‐confirmed patients admitted to intensive care units, internal medicine, or COVID‐19‐specific respiratory inpatient wards. COVID‐19‐associated mortality was defined in COVID‐19‐confirmed patients whose cause of death was ascribed to COVID‐19 or its complications.

Study participants date of death was ascertained by linking the study cohort with the Ministry of Interior registry. All study participants were followed up from the onset of the pandemic in Israel or the date of drug initiation, whichever occurs later, until October 2, 2020, drug discontinuation, death, or fulfilling the study outcomes, whichever occurs earlier.

### 2.5 Covariates

Outcome measures were adjusted for the following comorbid conditions: chronic obstructive pulmonary disease (COPD), chronic renal failure (CRF), diabetes mellitus, ischemic heart disease (IHD), hypertension, hyperlipidemia, and malignancies, all of which were evidenced to project worse prognosis of COVID‐19.^23^, ^24^, ^25^, ^26^, ^27^ COVID‐19 outcomes were additionally adjusted for smoking owing to the association of the latter with worse outcomes of COVID‐19.^23^, ^24^ The chronic registry of CHS was utilized to identify comorbidities of eligible patients prior to the development of COVID‐19.

### 2.6 Statistical analysis

The comparison between different variables was performed utilizing the chi‐square test and *t* test for categorical and continuous variables, respectively. Incidence rates of outcomes were calculated and expressed as the number of events per 1000 person‐years. Hazard ratios (HR)s for the risk of incident outcomes were obtained by the use of the Cox regression model. Two‐tailed p‐values less than 0.05 were considered as statistically significant. All statistical analyses were performed using the SPSS software, version 25 (SPSS, Armonk, NY: IBM Corp).

## 3 RESULTS

The current study included 1943, 1929, 348, and 1892 patients with psoriasis treated by TNFi, methotrexate, ustekinumab, and acitretin during the pandemic, respectively. Relative to patients treated by methotrexate, those treated by TNFi were younger at the onset of the pandemic, had a male predominance, and a lower frequency of COPD, diabetes mellitus, hypertension, hyperlipidemia, ischemic heart disease, and malignancy. The baseline characteristics of study participants are delineated in Table 1.

**TABLE 1:** Descriptive characteristics of the study population

Characteristic	TNF inhibitors (*N* = 1943)	Methotrexate (*N* = 1929)	Ustekinumab (*N* = 348)	Acitretin (*N* = 1892)
Age at the onset of pandemic, years				
Mean (SD)	48.8 (16.4)	58.6 (17.4)	50.8 (17.3)	56.4 (16.2)
Age at the onset of the disease, years				
Mean (SD)	39.2 (16.1)	49.6 (16.8)	40.4 (17.0)	47.7 (15.9)
Sex, *n* (%)				
Male	1116 (57.4%)	927 (48.1%)	196 (56.3%)	1231 (65.1%)
Female	827 (42.6%)	1002 (51.9%)	152 (43.7%)	661 (34.9%)
Ethnicity, *n* (%)				
Jews	1592 (81.9%)	1568 (81.3%)	308 (88.5%)	1502 (79.4%)
Arabs	351 (18.1%)	361 (18.7%)	40 (11.5%)	390 (20.6%)
Smoking, *n* (%)	900 (46.3%)	883 (45.8%)	209 (60.1%)	1029 (54.4%)
COPD, *n* (%)	60 (3.1%)	102 (5.3%)	19 (5.5%)	97 (5.1%)
Diabetes mellitus, *n* (%)	343 (17.7%)	549 (28.5%)	79 (22.7%)	464 (24.5%)
Hypertension, *n* (%)	458 (23.6%)	756 (39.2%)	114 (32.8%)	663 (35.0%)
Hyperlipidemia, *n* (%)	905 (46.6%)	1172 (60.8%)	194 (55.7%)	1103 (58.3%)
Ischemic heart disease, *n* (%)	173 (8.9%)	299 (15.5%)	40 (11.5%)	233 (12.3%)
Malignancy, *n* (%)	190 (9.8%)	358 (18.6%)	42 (12.1%)	257 (13.6%)
Chronic renal failure, *n* (%)	62 (3.2%)	66 (3.4%)	22 (6.3%)	103 (5.4%)

Abbreviations: BMI, body mass index; *n*, number; SD, SD.

### 3.1 Primary analysis comparing the risk of COVD‐19 outcomes associated with TNFi relative to methotrexate

The incidence rate of COVID‐19 infection, COVID‐19‐associated hospitalization, and COVID‐19‐associated mortality in the TNFi group was calculated at 35.8 (95% CI, 26.1‐47.9), 0.8 (95% CI, 0.0‐4.2), and 0.0 per 1000 person‐years, respectively. The corresponding incidence rates in the methotrexate group were 30.9 (95% CI, 23.6‐43.3), 10.3 (95% CI, 5.6‐17.5), and 0.9 (95% CI, 0.0‐4.2) per 1000 person‐years, respectively (Table 2).

**TABLE 2:** The risk of COVID‐19 and its complications among patients with psoriasis treated by TNF inhibitors compared to those treated by methotrexate

	COVID‐19 infection		COVID‐19‐associated hospitalization		COVID‐19‐associated mortality	
	TNF inhibitors (*N* = 1943)	Methotrexate (*N* = 1929)	TNF inhibitors (*N* = 1943)	Methotrexate (*N* = 1929)	TNF inhibitors (*N* = 1943)	Methotrexate (*N* = 1929)
**Follow‐up time, PY**	1174.5	1164.3	1179.5	1166.8	1180.0	1169.1
**Median follow‐up time, years (range)**	0.6 (0.0‐0.6)	0.6 (0.1‐0.6)	0.6 (0.0‐06)	0.6 (0.1‐0.6)	0.6 (0.0‐06)	0.6 (0.1‐0.6)
**Number of events**	42	36	1	12	0	1
**Incidence rate / 1000 PY (95% CI)**	35.8 (26.1–47.9)	30.9 (23.6–43.3)	0.8 (0.0–4.2)	10.3 (5.6–17.5)	0.0	0.9 (0.0–4.2)
**Unadjusted HR (95% CI) [p value]**	1.15 (0.74‐1.80) [0.528]	Reference	**0.08 (0.01‐0.63) [0.016]**	Reference	0.02 (0.00‐145 728.57) [0.610]	Reference
**Male‐specific HR (95% CI) [p value]**	1.01 (0.54‐1.89) [0.966]	Reference	0.01 (0.00‐2.76) [0.112]	Reference	0.13 (0.00‐135 668.50) [0.596]	Reference
**Female‐specific HR (95% CI) [p value]**	1.34 (0.71‐2.53) [0.369]	Reference	0.40 (0.04‐3.85) [0.428]	Reference	NA	Reference
**Age‐ and sex‐Adjusted HR (95% CI) [p value]**	1.07 (0.67‐1.70) [0.787]	Reference	**0.11 (0.01‐0.91) [0.040]**	Reference	NA [0.980]	Reference
**Fully adjusted HR (95% CI) [p value]** ^a^	1.07 (0.67‐1.71) [0.768]^a^	Reference	**0.10 (0.01‐0.82) [0.031]** ^a^	Reference	NA [0.964]^a^	Reference

Abbreviations: CI, confidence interval; HR, hazard ratio; *n*, number; NA, non‐applicable; PY, person‐year.

*Note*: **Bold,** significant value.

^a^ ‐Multivariate logistic regression model adjusting for age, sex, COPD, CRF, IHD, HTN, hyperlipidemia, obesity, malignancy, diabetes mellitus, smoking.

While the risk of COVID‐19 infection and COVID‐19‐associated mortality was comparable between the two groups, patients treated with TNFi exhibited a significantly decreased multivariate risk of COVID‐19‐associated hospitalization (fully‐adjusted HR, 0.10; 95% CI, 0.01‐0.82; p = 0.031; Table 2).

In a sensitivity analysis, we estimated the risk of the aforementioned outcomes in patients under adalimumab (n = 1166; Table S1), etanercept (n = 643; Table S2), and infliximab (n = 176; Table S3) relative to psoriasis patients managed by methotrexate. While the incidence rate of COVID‐19‐associated hospitalization and mortality was numerically lower among patients managed by each one of the three drugs, the HRs of the aforementioned outcomes fell out of significance. Of note, 42 patients were treated by more than a single agent during the course of the pandemic.

### 3.2 Secondary analyses comparing the risk of COVD‐19 outcomes associated with TNFi relative to ustekinumab and acitretin

The first secondary analysis aimed to compare patients managed by TNFi (*n* = 1943) and ustekinumab (*n* = 348). TNFi was associated with a significantly decreased risk of COVID‐19‐associated hospitalization (fully‐adjusted HR, 0.04; 95% CI, 0.00‐0.64; p = 0.022). The risk of COVID‐19 infection and COVID‐19‐associated mortality did not differ between the two subgroups (Table 3).

**TABLE 3:** The risk of COVID‐19 and its complications among patients with psoriasis treated by TNF inhibitors compared to those treated by ustekinumab

	COVID‐19 infection		COVID‐19‐associated hospitalization		COVID‐19‐associated mortality	
	TNF inhibitors (*N* = 1943)	Ustekinumab (*N* = 348)	TNF inhibitors (*N* = 1943)	Ustekinumab (*N* = 348)	TNF inhibitors (*N* = 1943)	Ustekinumab (*N* = 348)
**Follow‐up time, PY**	1174.5	209.3	1179.5	210.1	1180.0	210.3
**Median follow‐up time, years (range)**	0.6 (0.0–0.6)	0.6 (0.0‐0.6)	0.6 (0.0‐06)	0.6 (0.0‐0.6)	0.6 (0.0‐06)	0.6 (0.0‐0.6)
**Number of events**	42	7	1	3	0	1
**Incidence rate / 1000 PY (95% CI)**	35.8 (26.1–47.9)	35.4 (25.8‐47.6)	0.8 (0.0‐4.2)	14.3 (3.6‐38.9)	0.0	4.8 (0.2‐23.5)
**Unadjusted HR (95% CI) [p value]**	1.07 (0.48‐2.38) [0.874]	Reference	**0.06 (0.01‐0.57) [0.014]**	Reference	NA [0.783]	Reference
**Male‐specific HR (95% CI) [p value]**	0. 96 (0.33‐2.79) [0.942]	Reference	NA [0.648]	Reference	NA [0.790]	Reference
**Female‐specific HR (95% CI) [p value]**	1.21 (0.36‐4.08) [0.755]	Reference	NA [0.786]	Reference	NA	Reference
**Age‐ and sex‐Adjusted HR (95% CI) [P value]**	1.07 (0.48–2.38) [0.872]	Reference	**0.08 (0.01‐0.79) [0.030]**	Reference	NA [0.972]	Reference
**Fully adjusted HR (95% CI) [p value]** ^a^	1.07 (0.48‐2.40) [0.866]^a^	Reference	**0.04 (0.00‐0.64) [0.022]** ^a^	Reference	1.00 (0.01‐249.06) [1.000]^a^	Reference

Abbreviations: CI, confidence interval; HR, hazard ratio; *n*, number; NA, non‐applicable; PY, person‐year.

*Note*: **Bold,** significant value.

^a^ ‐Multivariate logistic regression model adjusting for age, sex, COPD, CRF, IHD, HTN, hyperlipidemia, obesity, malignancy, diabetes mellitus, smoking.

**FIGURE 1:**
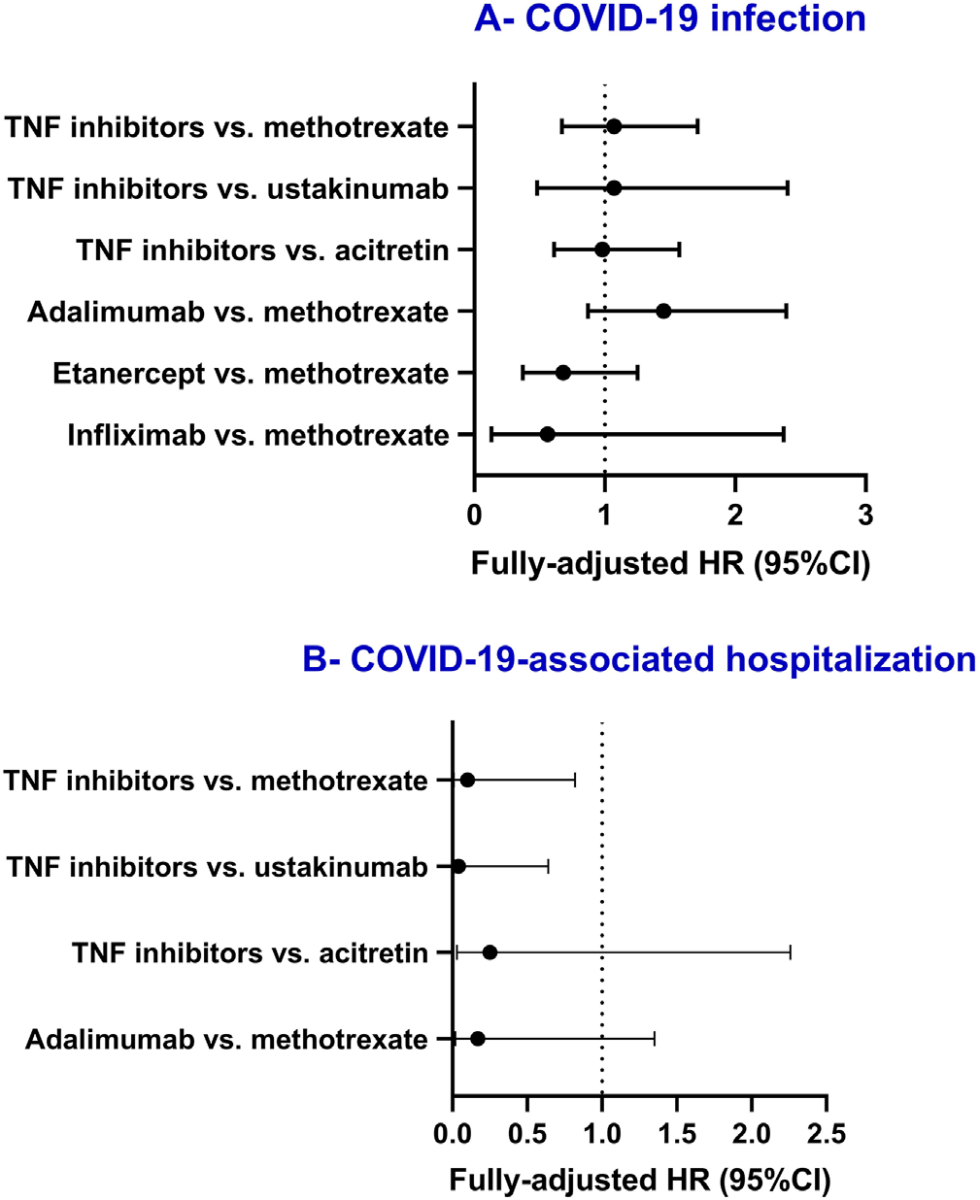
Hazard ratios (HRs) of COVID‐19 infection and COVID‐19‐associated hospitalization among patients under different treatments. Fully‐adjusted HR were utilized excluding COVID‐19‐associated mortality for TNFi versus methotrexate, where unadjusted HR (95% confidence interval [CI]) were demonstrated given that the adjusted model yielded wide range CI, which is not well graphically demonstrable

The second secondary analysis evaluated the differential risk of TNFi (*n* = 1869) as compared to acitretin (*n* = 1892). Out of the original subgroup of patients exposed to TNFi, 74 patients were excluded in the current analysis since they were concomitantly treated by acitretin. The risk of the three COVID‐19 outcomes of interest was comparable between the two subgroups (Table 4). Figure 1 graphically summarizes the main outcome measure of the current study.

**TABLE 4:** The risk of COVID‐19 and its complications among patients with psoriasis treated by TNF inhibitors compared to those treated by acitretin

	COVID‐19 infection		COVID‐19‐associated hospitalization		COVID‐19‐associated mortality	
	TNF inhibitors (*N* = 1869)^b^	Acitretin (*N* = 1892)	TNF inhibitors (*N* = 1869)^b^	Acitretin (*N* = 1892)	TNF inhibitors (*N* = 1869)^b^	Acitretin (*N* = 1892)
**Follow‐up time, PY**	1129.7	1143.9	1134.8	1148.5	1135.0	1148.9
**Median follow‐up time, years (range)**	0.6 (0.0–0.6)	0.6 (0.1–0.6)	0.6 (0.0‐0.6)	0.6 (0.1‐0.6)	0.6 (0.0‐0.6)	0.6 (0.1‐0.6)
**Number of events**	40	36	1	4	0	1
**Incidence rate/1000 PY (95% CI)**	35.4 (25.6‐47.7)	31.5 (22.4‐43.1)	0.8 (0.0‐4.3)	3.5 (1.1‐8.4)	0	0.9 (0.0–4.3)
**Unadjusted HR (95% CI) [p value]**	1.13 (0.72‐1.77) [0.609]	Reference	0.25 (0.03‐2.26) [0.218]	Reference	0.02 (0.00‐148 974.44) [0.611]	Reference
**Male‐specific HR (95% CI) [p value]**	1.15 (0.63‐2.11) [0.643]	Reference	0.02 (0.00‐194.97) [0.395]	Reference	0.02 (0.00‐179 172.91) [0.623]	Reference
**Female‐specific HR (95% CI) [p value]**	1.04 (0.53‐2.05) [0.902]	Reference	0.82 (0.05‐13.16) [0.890]	Reference	NA	Reference
**Age‐ and sex‐Adjusted HR (95% CI) [p value]**	0.99 (0.62‐1.59) [0.974]	Reference	0.29 (0.03‐2.79) [0.289]	Reference	NA [0.978]	Reference
**Fully‐adjusted HR (95% CI) [p value]** ^a^	0.98 (0.61‐1.57) [0.933]	Reference	1.00 (0.16–6.16) [0.100]	Reference	1.00 (0.02‐58.29) [1.000]	Reference

Abbreviations: CI, confidence interval; HR, hazard ratio; *n*, number; NA, non‐applicable; PY, person‐year.

*Note*: **Bold,** significant value.

^a^ ‐Multivariate logistic regression model adjusting for age, sex, COPD, CRF, IHD, HTN, hyperlipidemia, obesity, malignancy, diabetes mellitus, smoking.

^b^ All 74 patients who were concomitantly managed by TNFi and acitretin at the onset of the pandemic were excluded.

## 4 DISCUSSION

The current study provides the first population‐based estimate of the influence exerted by TNFi on the outcomes of COVID‐19 among patients with psoriasis. Relative to methotrexate and ustekinumab, TNFi conferred significant protection against the need for COVID‐associated hospitalization. The incidence of COVID‐19‐associated mortality was numerically lower among patients treated by TNFi as compared to all other reference groups, albeit without reaching the level of significance.

Evidence has accumulated from several observational studies to suggest a putative protective effect of TNFi against COVID‐19 complications. Based on data from the SECURE‐IBD registry, which followed patients with IBD who developed COVID‐19, TNFi therapy was inversely associated with death or hospital admission for COVID‐19 (adjusted odds ratio [OR] 0.60; 95% CI 0·38‐0·96).^14^ Similarly, in a global registry of 600 patients with rheumatic diseases and COVID‐19, biologic agents, 52% of which were TNFis, were associated with a decreased risk of COVID‐19‐associated hospitalization (OR, 0.46; 95% CI 0.22‐0.93).^4^ In a recent large‐scale study, 101 random patients with COVID‐19 who were recently exposed to TNFi were compared with 101 patients with COVID‐19 without TNFi exposure. In this study, TNFi did not impose an increased risk either of hospitalization nor mortality.^28^

In psoriasis, however, previous research focused on the general risk of COVID‐19 and its outcomes under immunosuppressive and immunomodulatory drugs without dissecting the specific risk attributed to TNFi. A monocentric study from Lombardy reported five hospitalizations and no deaths among 1193 psoriatic patients treated with biologics or immunosuppressive agents. The likelihood of COVID‐19‐associated hospitalization was increased relative to the general domestic population (OR, 3.59; 95% CI,1.49‐8.63).^19^ Most other studies disclosed that immunomodulatory treatment in psoriasis conferred a minimal risk of fatality and severe disease necessitating admission. No cases of COVID‐19‐associated hospitalization or mortality were detected in a retrospective study from Northern Italy following 980 psoriasis patients undergoing biologic treatment between February and April 2020.^16^ Congruently, a multicenter Italian study followed 5206 biologics‐treated patients with psoriasis for 2 months and identified no cases of mortality and five cases of admission to hospitals.^17^ In an international registry‐based study encompassing 374 patients with psoriasis and COVID‐19, hospitalization was more frequent in patients under nonbiologic systemic therapy than in those under biologics (OR, 2.84; 95% CI, 1.31‐6.18).^29^ Consistent findings emerged in a French multicenter study recruiting 1418 patients receiving systemic treatment, including biologics, methotrexate, cyclosporine, acitretin, and apremilast. This study detected no deaths and only 5 (0.4%) patients with severe COVID‐19 warranting hospitalization, of whom three (60.0%) had severe preexisting comorbidities.^30^ Consistent findings of negligible risk of COVID‐19 related complications were conveyed by other observational studies focusing on patients with psoriasis treated by immunomodulatory agents.^15^, ^18^

Our study denoted that the risk of acquiring COVID‐19 infection was not significantly different when comparing patients managed by TNFi and other systemic agents. This observation accords, at least in part, with previous studies demonstrating a comparable risk of COVID‐19 among patients with psoriasis managed by systemic drugs relative to the general population.^15^, ^31^, ^32^The latter lend weight to the assumption that susceptibility to the infection stems mainly from whether patients get exposed to the pathogen, adhere to social distancing, and follow safety instructions. The more intriguing question, however, relates to the COVID‐19 course patients under TNFi and other systemic drugs follow.

We found that TNFi exerted a protective role against COVID‐19‐associated hospitalization relative to methotrexate and ustekinumab. This favorable role embodied by TNFi is conceivable in light of the fact that this treatment leads to downregulation of TNF alongside other pro‐inflammatory mediators, including IL‐1, IL‐6, VEGF, and granulocyte‐macrophage colony‐stimulating factor within 24 h.^33^, ^34^ Since these mediators are implicated in the inflammation‐driven capillary leak in COVID‐19, TNFi might hypothetically ameliorate lung injury and prevent severe complications and mortality. It is noteworthy that patients under TNFi demonstrated a lower incidence rate of COVID‐associated mortality in each one of the analyses, but the latter was not of statistical significance, mainly due to the small sample size of patients with positive outcomes. Larger study populations and more extended duration of follow‐up will render futuristic studies more statistically powered to identify a significant difference between the subgroups.

The favorable effect of TNFi in COVID‐19 is supported both by observational clinical studies and biological mechanistic plausibility. This effect led some authors to advocate trialing this drug class as a putative therapeutic intervention in COVID‐19.^12^, ^35^ Surprisingly, very few studies are currently ongoing to evaluate TNFi therapy in COVID‐19. Until then, caution should be practiced in the interpretation of the findings originating from observational studies. In the current study, as well as in other well‐designed observational studies,^4^, ^14^ comparators were other patients with psoriasis or immune‐related diseases. These patients might hypothetically respond distinctly to COVID‐19 owing to chronic changes in their immune system and previous immunomodulatory treatments. Whether the results stemming from the observational studies are generalizable to the general population, therefore, remains to be delineated by randomized controlled trials. On the other hand, the current findings are sufficiently robust to suggest the continuation of TNFi drug and even initiation of this drug class during the pandemic.

The current study provides the first population‐based estimate of COVID‐19 outcomes among patients with psoriasis treated by TNFi. In the current study, multiple analyses were undertaken to compare the influence of this drug class with different agents and to provide clinicians with a broad perspective regarding its relative safety. The study population is large, and the length of follow‐up is longer than most published studies. The main limitation of the study emanates from the small number of cases with positive outcomes in the sensitivity analysis and mortality analyses. The latter impedes obtaining outcomes with statistical significance and yield wide margin CI. Further studies with even longer follow‐up are necessary to subdue this drawback. Moreover, we were unable to evaluate the outcomes of COVID‐19 under less frequent TNFi agents like certolizumab and golimumab. Outcomes of patients with psoriasis in the current study were not compared with those of the general Israeli population. The latter represents another prominent limitation of our study. The study was additionally limited by the small sample size of the ustekinumab comparison group.

In conclusion, the current large‐scale population‐based study revealed that TNFi treatment during the pandemic was associated with a decreased risk of COVID‐19 hospitalization as compared to methotrexate and ustekinumab. These findings substantiate the approach suggesting to avoid preventive cessation of TNFi treatment unless indicated individually by the patient's clinical data, comorbidities, or specific risk factors. In moderate‐to‐severe plaque psoriasis necessitating systemic treatment, TNFi should be positively considered. Further studies with longer follow‐up are warranted to provide a broader insight into the influence of this drug class on COVID‐19.

## CONFLICT OF INTEREST

Arnon D. Cohen served as an advisor, investigator, or speaker for Abbvie, BI, Dexcel Pharma, Janssen, Novartis, Perrigo, Pfizer, and Rafa. DTB received a research grant from Pfizer. None of the other authors have any conflicts of interest to declare.

## Supporting information


Appendix S1: Supporting information

## Data Availability

The datasets generated during and/or analysed during the current study are available from the corresponding author on reasonable request.

## References

[1] Shi Y , Wang Y , Shao C , et al. COVID‐19 infection: the perspectives on immune responses. Cell Death Differ. 2020;27(5):1451‐1454. 10.1038/s41418-020-0530-3. 32205856 PMC7091918

[2] Vabret N , Britton GJ , Gruber C , et al. Immunology of COVID‐19: current state of the science. Immunity. 2020;52(6):910‐941. 10.1016/j.immuni.2020.05.002. 32505227 PMC7200337

[3] Pablos JL , Galindo M , Carmona L , et al. Clinical outcomes of hospitalised patients with COVID‐19 and chronic inflammatory and autoimmune rheumatic diseases: a multicentric matched cohort study. Ann Rheum Dis. 2020;79(12):1544‐1549. 32796045 10.1136/annrheumdis-2020-218296

[4] Gianfrancesco M , Hyrich KL , Hyrich KL , et al. Characteristics associated with hospitalisation for COVID‐19 in people with rheumatic disease: data from the COVID‐19 global rheumatology Alliance physician‐reported registry. Ann Rheum Dis. 2020;79(7):859‐866. 10.1136/annrheumdis-2020-217871. 32471903 PMC7299648

[5] D'Silva KM , Serling‐Boyd N , Wallwork R , et al. Clinical characteristics and outcomes of patients with coronavirus disease 2019 (COVID‐19) and rheumatic disease: a comparative cohort study from a US hot spot. Ann Rheum Dis. 2020;79(9):1156‐1162. 10.1136/annrheumdis-2020-217888. 32457048 PMC7456555

[6] Ansarin K , Taghizadieh A , Safiri S , et al. COVID‐19 outcomes in patients with systemic autoimmune diseases treated with immunomodulatory drugs. Ann Rheum Dis. 2020. 10.1136/annrheumdis-2020-218737. 32759256

[7] Emmi G , Bettiol A , Mattioli I , et al. SARS‐CoV‐2 infection among patients with systemic autoimmune diseases. Autoimmun Rev. 2020;19(7):102575. 10.1016/j.autrev.2020.102575. 32376395 PMC7200134

[8] Liu M , Gao Y , Zhang Y , Shi S , Chen Y , Tian J . The association between severe or dead COVID‐19 and autoimmune diseases: a systematic review and meta‐analysis. J Clean Prod. 2020;81:e93‐e95. 10.1016/j.jinf.2020.05.065. PMC7264926 32502509

[9] Fredi M , Cavazzana I , Moschetti L , et al. COVID‐19 in patients with rheumatic diseases in northern Italy: a single‐Centre observational and case–control study. Lancet Rheumatol. 2020;2(9):e549‐e556. 10.1016/S2665-9913(20)30169-7. 32838307 PMC7302769

[10] Macaluso FS , Orlando A . COVID‐19 in patients with inflammatory bowel disease: a systematic review of clinical data. Dig Liver Dis. 2020;52(11):1222‐1227. 10.1016/j.dld.2020.09.002. 32928672 PMC7474894

[11] Urquhart L . Top product forecasts for 2020. Nat Rev Drug Discov. 2020;19(2):86. 10.1038/d41573-020-00011-5. 32020077

[12] Robinson PC , Richards D , Tanner HL , Feldmann M . Accumulating evidence suggests anti‐TNF therapy needs to be given trial priority in COVID‐19 treatment. Lancet Rheumatol. 2020;2(11):e653‐e655. 10.1016/S2665-9913(20)30309-X. 33521660 PMC7832144

[13] Bongartz T , Sutton AJ , Sweeting MJ , Buchan I , Matteson EL , Montori V . Anti‐TNF antibody therapy in rheumatoid arthritis and the risk of serious infections and malignancies: systematic review and meta‐analysis of rare harmful effects in randomized controlled trials. JAMA. 2006;295(19):2275‐2285. 10.1001/jama.295.19.2275. 16705109

[14] Brenner EJ , Ungaro RC , Gearry RB , et al. Corticosteroids, but not TNF antagonists, are associated with adverse COVID‐19 outcomes in patients with inflammatory bowel diseases: results from an international registry. Gastroenterology. 2020;159(2):481‐491.e3. 10.1053/j.gastro.2020.05.032. 32425234 PMC7233252

[15] Piaserico S , Gisondi P , Cazzaniga S , Naldi L . Lack of evidence for an increased risk of severe COVID‐19 in psoriasis patients on biologics: a cohort study from Northeast Italy. Am J Clin Dermatol. 2020;21(5):749‐751. 10.1007/s40257-020-00552-w. 32812188 PMC7433672

[16] Gisondi P , Zaza G , Del Giglio M , Rossi M , Iacono V , Girolomoni G . Risk of hospitalization and death from COVID‐19 infection in patients with chronic plaque psoriasis receiving a biologic treatment and renal transplant recipients in maintenance immunosuppressive treatment. J Am Acad Dermatol. 2020;83(1):285‐287. 10.1016/j.jaad.2020.04.085. 32330632 PMC7194926

[17] Gisondi P , Facheris P , Dapavo P , et al. The impact of the COVID‐19 pandemic on patients with chronic plaque psoriasis being treated with biological therapy: the northern Italy experience. Br J Dermatol. 2020;183(2):373‐374. 10.1111/bjd.19158. 32343839 PMC7267283

[18] Burlando M , Carmisciano L , Cozzani E , Parodi A . A survey of psoriasis patients on biologics during COVID‐19: a single Centre experience. J Dermatol Treat. 2020;1. 10.1080/09546634.2020.1770165. 32406283

[19] Damiani G , Pacifico A , Bragazzi NL , Malagoli P . Biologics increase the risk of SARS‐CoV‐2 infection and hospitalization, but not ICU admission and death: real‐life data from a large cohort during red‐zone declaration. Dermatol Ther. 2020;33(5):e13475. 10.1111/dth.13475. 32356577 PMC7261990

[20] Cohen AD , Dreiher J , Regev‐Rosenberg S , et al. The quality indigators program in Clalit Health Services: the first decade. Harefuah. 2010;149(4):204–209. 20812490

[21] Dogra S , Mahajan R . Systemic methotrexate therapy for psoriasis: past, present and future. Clin Exp Dermatol. 2013;38(6):573‐588. 10.1111/ced.12062. 23837932

[22] Kalb RE , Fiorentino DF , Lebwohl MG , et al. Risk of serious infection with biologic and systemic treatment of psoriasis: results from the psoriasis longitudinal assessment and registry (PSOLAR). JAMA Dermatol. 2015;151(9):961‐969. 10.1001/jamadermatol.2015.0718. 25970800

[23] Zheng Z , Peng F , Xu B , et al. Risk factors of critical & mortal COVID‐19 cases: a systematic literature review and meta‐analysis. J Infect. 2020;81(2):e16‐e25. 10.1016/j.jinf.2020.04.021. PMC7177098 32335169

[24] Zhao Q , Meng M , Kumar R , et al. The impact of COPD and smoking history on the severity of COVID‐19: a systemic review and meta‐analysis. J Med Virol. 2020;92(10):1915‐1921. 10.1002/jmv.25889. 32293753 PMC7262275

[25] Ali H , Daoud A , Mohamed MM , et al. Survival rate in acute kidney injury superimposed COVID‐19 patients: a systematic review and meta‐analysis. Ren Fail. 2020;42(1):393‐397. 10.1080/0886022X.2020.1756323. 32340507 PMC7241495

[26] Huang I , Lim MA , Pranata R . Diabetes mellitus is associated with increased mortality and severity of disease in COVID‐19 pneumonia—a systematic review, meta‐analysis, and meta‐regression: diabetes and COVID‐19. Diabetes Metab Syndr Clin Res Rev. 2020;14(4):395‐403. 10.1016/j.dsx.2020.04.018. PMC7162793 32334395

[27] Mehta V , Goel S , Kabarriti R , et al. Case fatality rate of cancer patients with COVID‐19 in a New York hospital system. Cancer Discov. 2020;10(7):935‐941. 10.1158/2159-8290.CD-20-0516. 32357994 PMC7334098

[28] Yousaf A , Gayam S , Feldman S , Zinn Z , Kolodney M . Clinical outcomes of COVID‐19 in patients taking tumor necrosis factor inhibitors or methotrexate: a multicenter research network study. J Am Acad Dermatol. 2021;84(1):70‐75. 10.1016/j.jaad.2020.09.009. 32926977 PMC7484805

[29] Mahil SK , Dand N , Mason KJ , et al. Factors associated with adverse COVID‐19 outcomes in patients with psoriasis—insights from a global registry–based study. J Allergy Clin Immunol. 2021;147(1):60‐71. 10.1016/j.jaci.2020.10.007. 33075408 PMC7566694

[30] Fougerousse AC , Perrussel M , Bécherel PA , et al. Systemic or biologic treatment in psoriasis patients does not increase the risk of a severe form of COVID‐19. J Eur Acad Dermatol Venereol. 2020;34(11):e676‐e679. 10.1111/jdv.16761. 32564417 PMC7323155

[31] Baniandrés‐Rodríguez O , Vilar‐Alejo J , Rivera R , et al. Incidence of severe COVID‐19 outcomes in psoriatic patients treated with systemic therapies during the pandemic: a Biobadaderm cohort analysis. J Am Acad Dermatol. 2021;84(2):513‐517. 10.1016/j.jaad.2020.10.046. 33122022 PMC7587130

[32] Vispi M , Corradin T , Peccianti C , et al. Psoriasis, biological drugs and coronavirus disease 2019: real life experience of two Italian provinces. Dermatol Rep. 2020;12(1):8642. 10.4081/dr.2020.8642. PMC7336268 32655846

[33] Elliott MJ , Maini RN , Feldmann M , et al. Treatment of rheumatoid arthritis with chimeric monoclonal antibodies to tumor necrosis factor α. Arthritis Rheum. 1993;36(12):1681‐1690. 10.1002/art.1780361206. 8250987

[34] Charles P , Elliott MJ , Davis D , et al. Regulation of cytokines, cytokine inhibitors, and acute‐phase proteins following anti‐TNF‐alpha therapy in rheumatoid arthritis. J Immunol. 1999;163(3):1521‐1528. 10415055

[35] Feldmann M , Maini RN , Woody JN , et al. Trials of anti‐tumour necrosis factor therapy for COVID‐19 are urgently needed. Lancet. 2020;395(10234):1407‐1409. 10.1016/S0140-6736(20)30858-8. 32278362 PMC7158940

