# Supplementary material for: Tumor necrosis factor inhibitors are associated with a decreased risk of COVID‐19‐associated hospitalization in patients with psoriasis—A population‐based cohort study
Source: Dermatol Ther. 2021 Jun 5;34(4):e15003. doi: 10.1111/dth.15003 (PMC8209905; doi:10.1111/dth.15003)
Supplement: Supplementary file 1 — Appendix S1: Supporting information [file DTH-34-0-s001.docx]

**Supplementary table 1-** The risk of COVID-19 and its complications among patients with psoriasis treated by adalimumab compared to those treated by methotrexate

|  | **COVID-19 infection** | | **COVID-19-associated hospitalization** | | **COVID-19-associated mortality** | |
| --- | --- | --- | --- | --- | --- | --- |
|  | **Adalimumab (N=1,166)** | **Methotrexate (N=1,929)** | **Adalimumab (N=1,166)** | **Methotrexate (N=1,929)** | **Adalimumab (N=1,166)** | **Methotrexate (N=1,929)** |
| **Follow-up time, PY** | 704.7 | 1,164.3 | 708.3 | 1,166.8 | 708.5 | 1,169.1 |
| **Median follow-up time, years (range)** | 0.6 (0.0-0.6) | 0.6 (0.1-0.6) | 0.6 (0.0-0.6) | 0.6 (0.1-0.6) | 0.6 (0.0-0.6) | 0.6 (0.1-0.6) |
| **Number of events** | 33 | 36 | 1 | 12 | 0 | 1 |
| **Incidence rate / 1000 PY (95% CI)** | 46.8 (32.8-65.0) | 30.9 (23.6-43.3) | 1.4 (0.1-7.0) | 10.3 (5.6-17.5) | 0.0 | 0.9 (0.0-4.2) |
| **Crude HR (95% CI) [P value]** | 1.51 (0.94-2.43) [0.085] | Reference | 0.14 (0.02-1.05) [0.056] | Reference | 0.02 (0.00-450,410.81) [0.660] | Reference |
| **Male-specific HR (95% CI) [P value]** | 1.17 (0.60-2.30) [0.646] | Reference | 0.02 (0.00-4.48) [0.156] | Reference | 0.02 (0.00-236826.80) [0.637] | Reference |
| **Female-specific HR (95% CI) [P value]** | **2.04 (1.05-3.96) [0.035]** | Reference | 0.72 (0.07-6.88) [0.772] | Reference | NA | Reference |
| **Age- and sex-Adjusted HR (95% CI) [P value]** | 1.42 (0.86-2.34) [0.174] | Reference | 0.20 (0.03-1.63) [0.133] | Reference | NA [0.982] | Reference |
| **Fully adjusted HR (95% CI) [P value]^a^** | 1.45 (0.87-2.39) [0.152]^a^ | Reference | 0.17 (0.02-1.35) [0.093]^a^ | Reference | NA [0.975]^a^ | Reference |

^a^-Multivariate logistic regression model adjusting for age, sex, COPD, CRF, IHD, HTN, hyperlipidemia, obesity, malignancy, diabetes mellitus, smoking

**Abbreviations:** n, Number; PY, person-year; HR, hazard ratio; CI, confidence interval; NA, non-applicable

**Bold:** significant value

**Supplementary table 2-** The risk of COVID-19 and its complications among patients with psoriasis treated by etanercept compared to those treated by methotrexate

|  | **COVID-19 infection** | | **COVID-19-associated hospitalization** | | **COVID-19-associated mortality** | |
| --- | --- | --- | --- | --- | --- | --- |
|  | **Etanercept (N=644)** | **Methotrexate (N=1,929)** | **Etanercept (N=643)** | **Methotrexate (N=1,929)** | **Etanercept (N=643)** | **Methotrexate (N=1,929)** |
| **Follow-up time, PY** | 388.2 | 1,164.3 | 390.0 | 1,166.8 | 390.0 | 1,169.1 |
| **Median follow-up time, years (range)** | 0.6 (0.0-0.6) | 0.6 (0.1-0.6) | 0.6 (0.0-0.6) | 0.6 (0.1-0.6) | 0.6 (0.0-0.6) | 0.6 (0.1-0.6) |
| **Number of events** | 9 | 36 | 0 | 12 | 0 | 1 |
| **Incidence rate / 1000 PY (95% CI)** | 23.2 (11.3-42.6) | 30.9 (23.6-43.3) | 0.0 | 10.3 (5.6-17.5) | 0.0 | 0.9 (0.0-4.2) |
| **Crude HR (95% CI) [P value]** | 0.75 (0.36-1.55) [0.434] | Reference | 0.03 (0.00-8.17) [0.223] | Reference | 0.03 (0.00-7,223,813.72) [0.725] | Reference |
| **Male-specific HR (95% CI) [P value]** | 1.05 (0.44-2.51) [0.916] | Reference | 0.03 (0.00-15.28) [0.271] | Reference | 0.03 (0.00-3971,987.17) [0.714] | Reference |
| **Female-specific HR (95% CI) [P value]** | 0.37 (0.44-2.51) [0.181] | Reference | 0.72 (0.00-3,171.86) [0.560] | Reference | NA | Reference |
| **Age- and sex-Adjusted HR (95% CI) [P value]** | 0.76 (0.42-1.38) [0.370] | Reference | NA [0.962] | Reference | NA [0.984] | Reference |
| **Fully adjusted HR (95% CI) [P value]^a^** | 0.68 (0.37-1.25) [0.213]^a^ | Reference | NA [0.970]^a^ | Reference | NA [0.981]^a^ | Reference |

^a^-Multivariate logistic regression model adjusting for age, sex, COPD, CRF, IHD, HTN, hyperlipidemia, obesity, malignancy, diabetes mellitus, smoking

**Abbreviations:** n, Number; PY, person-year; HR, hazard ratio; CI, confidence interval; NA, non-applicable

**Bold:** significant value

**Supplementary table 3-** The risk of COVID-19 and its complications among patients with psoriasis treated by infliximab compared to those treated by methotrexate

|  | **COVID-19 infection** | | **COVID-19-associated hospitalization** | | **COVID-19-associated mortality** | |
| --- | --- | --- | --- | --- | --- | --- |
|  | **Infliximab (N=176)** | **Methotrexate (N=1,929)** | **Infliximab (N=176)** | **Methotrexate (N=1,929)** | **Infliximab (N=176)** | **Methotrexate (N=1,929)** |
| **Follow-up time, PY** | 106.3 | 1,164.3 | 106.5 | 1,166.8 | 106.5 | 1,169.1 |
| **Median follow-up time, years (range)** | 0.6 (0.0-0.6) | 0.6 (0.1-0.6) | 0.6 (0.0-0.6) | 0.6 (0.1-0.6) | 0.6 (0.0-0.6) | 0.6 (0.1-0.6) |
| **Number of events** | 2 | 36 | 0 | 12 | 0 | 1 |
| **Incidence rate / 1000 PY (95% CI)** | 18.8 (3.2-62.2) | 30.9 (23.6-43.3) | 0.0 | 10.3 (5.6-17.5) | 0.0 | 0.9 (0.0-4.2) |
| **Crude HR (95% CI) [P value]** | 0.61 (0.15-2.51) [0.489] | Reference | 0.04 (0.00-352.90) [0.495] | Reference | NA[0.844] | Reference |
| **Male-specific HR (95% CI) [P value]** | 0.53 (0.07-3.98) [0.538] | Reference | 0.04 (0.00-790.86) [0.530] | Reference | NA [0.834] | Reference |
| **Female-specific HR (95% CI) [P value]** | 0.69 (0.09-5.20) [0.723] | Reference | 0.04 (0.00-8,898,266.11) [0.749] | Reference | NA | Reference |
| **Age- and sex-Adjusted HR (95% CI) [P value]** | 0.54 (0.13-2.28) [0.401] | Reference | NA [0.985] | Reference | NA [0.991] | Reference |
| **Fully adjusted HR (95% CI) [P value]^a^** | 0.56 (0.13-2.37) [0.432]^a^ | Reference | NA [0.985]^a^ | Reference | NA [0.987]^a^ | Reference |

^a^-Multivariate logistic regression model adjusting for age, sex, COPD, CRF, IHD, HTN, hyperlipidemia, obesity, malignancy, diabetes mellitus, smoking

**Abbreviations:** n, Number; PY, person-year; HR, hazard ratio; CI, confidence interval; NA, non-applicable

**Bold:** significant value
